# 1527. Assessment of Human Immunodeficiency Virus Testing in Underinsured Persons

**DOI:** 10.1093/ofid/ofad500.1362

**Published:** 2023-11-27

**Authors:** Andrew M Skinner

**Affiliations:** University of Utah, Holladay, Utah

## Abstract

**Background:**

Among those living with HIV, nearly 13% are unaware of their diagnosis and approximately 40% of new HIV infections are transmitted from those lacking a formal diagnosis. Thus, determination of populations at high risk of not having HIV testing completed are essential to reduce transmissions. A barrier to obtaining an HIV test within low-income individuals that do not qualify for Medicaid, or underinsured persons, is the rising cost of healthcare in the US. Thus, it is essential to determine if further resources should be dedicated to underinsured populations with the aim of increasing access to HIV testing.

**Methods:**

This is a retrospective cross-sectional study utilizing the 2021 Behavior Risk Factor Surveillance System conducted by the US CDC to determine if individuals that do not qualify for Medicaid yet live in a household with an income of >138% but ≤200% of the US federal poverty line (FPL) had increased odds of not having an HIV test when compared to individuals that live in a household with an income of ≤138% of the FPL, and thus qualify for Medicaid. A multivariable logistic regression model was created to determine the adjusted odds ratio for not having an HIV test in those who did not qualify for Medicaid.

**Results:**

This study analyzed data from 33,431 respondents that were age 18 – 64, living in a Medicaid expansion state, and had a household income of ≤200% the FPL. Among these persons, 65.1% of respondents qualified for Medicaid. Univariate analysis revealed that non-Medicaid eligible persons were more likely to not have an HIV test when compared to Medicaid eligible persons with 54% and 48.6% not having an HIV test, respectively. After adjusting for the confounding variables, persons who were not eligible for Medicaid had 1.26 (95% CI:1.21 - 1.32) times the odds of not having an HIV test compared to those who were eligible for Medicaid.(Table)

**Figure:**
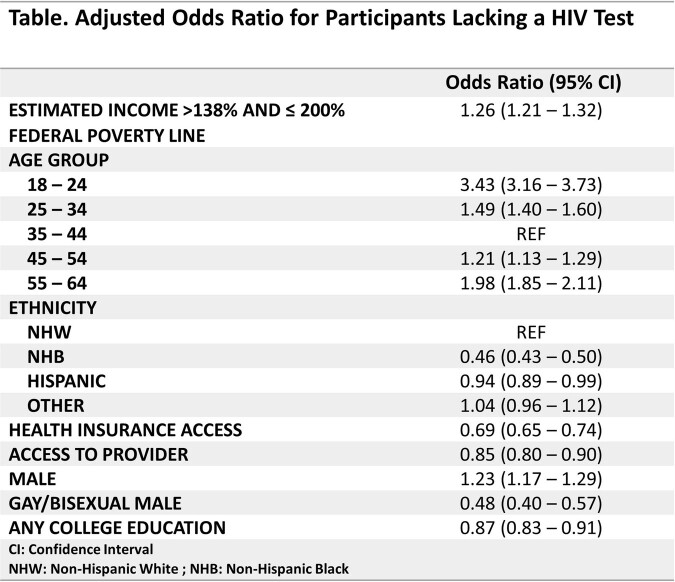

**Conclusion:**

After adjusting for confounding variables, the odds that a person did not have an HIV test were higher among individuals that lived in a household with an income that was >138% but ≤200% of the FPL. These data reveal that underinsured persons are a potential group that may benefit from further resource allocation to bolster HIV screening.

**Disclosures:**

**Andrew M. Skinner, MD**, Academy for Continued Healthcare Learning: Honoraria|American Society of Healthcare Pharmacists: Honoraria|Ferring Pharmaceuticals: Honoraria|MJH Life Sciences: Honoraria

